# Recent Advances in Antifouling Materials for Surface Plasmon Resonance Biosensing in Clinical Diagnostics and Food Safety

**DOI:** 10.3390/polym13121929

**Published:** 2021-06-10

**Authors:** Roberta D’Agata, Noemi Bellassai, Vanessa Jungbluth, Giuseppe Spoto

**Affiliations:** 1 Dipartimento di Scienze Chimiche, Università degli Studi di Catania, Viale Andrea Doria 6, I-95125 Catania, Italy; noemi.bellassai@unict.it (N.B.); vanessa.jungbluth@unict.it (V.J.); 2 Consorzio Interuniversitario “Istituto Nazionale Biostrutture e Biosistemi”, c/o Dipartimento di Scienze Chimiche, Università di Catania, Viale Andrea Doria 6, I-95125 Catania, Italy

**Keywords:** antifouling, surface plasmon resonance, clinical diagnosis, food safety, biosensing

## Abstract

Strategies to develop antifouling surface coatings are crucial for surface plasmon resonance (SPR) sensing in many analytical application fields, such as detecting human disease biomarkers for clinical diagnostics and monitoring foodborne pathogens and toxins involved in food quality control. In this review, firstly, we provide a brief discussion with considerations about the importance of adopting appropriate antifouling materials for achieving excellent performances in biosensing for food safety and clinical diagnosis. Secondly, a non-exhaustive landscape of polymeric layers is given in the context of surface modification and the mechanism of fouling resistance. Finally, we present an overview of some selected developments in SPR sensing, emphasizing applications of antifouling materials and progress to overcome the challenges related to the detection of targets in complex matrices relevant for diagnosis and food biosensing.

## 1. Introduction

One of the most challenging goals in SPR sensor development is to fabricate surfaces capable of highly selective and specific interactions with the analyte in a complex fluid such as human serum/plasma or heterogeneous food matrixes [1]. The critical issue of such a sensor system is the ability to prevent or to repel weakly adhered biomolecules after their non-specific adsorption, referred to as the “fouling effect” [2], with a consequent reduction of the assay sensitivity, selectivity, and reproducibility [3]. The surfaces of these systems have to be finely tuned to achieve desired interactions with media in which they operate and in which the abundance of target molecules is often prohibitive [4]. Several procedures, such as dilution, filtering, or centrifugation, can contribute to the antifouling phenomenon by reducing the concentration of non-target molecules [5]. At the same time, these sample handling processes could lead to considerable loss of analytes, especially when dealing with early detection of diseases or foodborne marker screening, by increasing more error sources and possibly performing less accurate analytical results [6].

Most antifouling physical strategies rely on concepts that prevent or remove the “foulant” adhesion to the surfaces after it has been attached. Traditional surface blocking non-reactive compounds (e.g., bovine serum albumin (BSA), Tween 20 surfactant, and commercially available mixtures) could be added to the sample or in the running buffer to minimize non-specific adsorption [7]. Although some of them provide robust antifouling strategies, even successful for a variety of targets within human body fluids [8,9], they often suffer from slow response times, diffusion-limited kinetics, and degradation in consequence of the removal of the foulant, which may negatively affect the target detection.

Other hybrid approaches such as membrane cloaking [10], pretreating the surface with blank serum [11], adding dextran to the sample [12], and depleting the sample in background protein [13], succeeded to minimize the impact of the fouling effect but with numerous drawbacks in terms of sample handling or complexity.

Chemical strategies based on the modification of sensing surfaces with antifouling polymers cover a rather large group of molecules (e.g., PEG-based materials, zwitterionic polymers, and polysaccharide-based hydrogels), which have proved to be the best option still, as they are the most versatile and frequently used approaches [14,15]. Ideally, the interfacial layer of the metal-coated SPR sensors should present: (i) High binding capacity and sensitivity in detecting a low amount of target in undiluted complex media, (ii) easily functionalizable chemical groups for ligand attachment, and (iii) no inhibition of analyte binding, thus ensuring the overall sensing performance at the end.

Over the past three decades, the advance of SPR biosensing has been mainly focused on achieving clinically relevant thresholds for ultrasensitive target detection [16], while the design of antifouling coatings, especially for food testing [17], has been scarcely investigated [18]. Different approaches have made use of novel nanomaterials and nanostructures (e.g., gold nanoparticles. AuNPs) [19,20], also benefiting from microfluidics [21] to optimize the SPR assay sensitivity [22,23] for a wide range of biomarkers [24] in an easily accessible and minimally invasive way, avoiding multistep, costly, and time-consuming procedures. These possibilities are very promising for point-of-care applications and have been reviewed elsewhere [25].

Until now, the analytical performances of SPR and SPR imaging (SPRi) biosensors enable markers detection at the low femtomolar (fM) concentration [26] in complex biofluids as well [27]. These platforms offer a challenging, non-invasive strategy towards the liquid biopsy approach [28] that requires higher performances to identify ultra-low-concentrated markers under increased fouling conditions [29].

In this context, many efforts need to be directed towards designing antifouling materials that should provide surface coatings suitable for real-world applications in undiluted complex matrices with no unaltered sensitive and selective signal response [30].

It is worth pointing out that the distance between surface and target plays a key role in SPR biosensors. Hence, in the sensor design, the thickness of the layer must also be considered [31]. Usually, the thickness of the antifouling layer ranges from 15 and about 70 nm, thus introducing a significant constraint due to loss of sensitivity. Indeed, a reasonable estimate of the decay length (ld) (37% of SPR wavelength) [32] of the evanescent plasmonic wave, around a few hundred nanometers, poses limits to the thickness of the layer beyond the metallic surface for which sensitive detection of the target can be obtained.

Particular attention should be deserved in the strategy implemented to overcome the above-discussed requirements when designing antifouling materials for SPR biosensing.

This review highlights advances in the performance of SPR biosensors for the determination of markers in both diagnostics and food science applications. After discussing the importance of achieving an optimal resilience to fouling and giving a non-exhaustive landscape of antifouling materials type, we gave particular attention to novel antifouling approaches succeeding in detecting relevant biomarkers using detection formats debated in the final two sections of diagnostics and food sensing.

## 2. Why Are the Antifouling Strategies So Essential for SPR Detection in Clinical Diagnostics and Food Safety?

The needs concerning the sensitivity, selectivity, and limit of detection (LOD) are different when dealing with food safety samples than those in health diagnosis. The analytes of interest in food samples may be typically small molecules such as chemicals, toxins, pesticides, and heavy metals [33]. In addition, proteins, nucleic acids, bacteria, and vesicles are often targeted as relevant biomarkers for food control or clinical diagnosis [34].

The development of an antifouling layer should consider both the analyte size as well as the detection format. For larger molecules, such as proteins, it needs to consider the capability to catch the analyte into the proximity of the active region of the SPR sensor surface for higher sensitivity. Moreover, the bigger a protein, the more likely it is to bear multiple adhesion sites, therefore quickly adsorb to a surface. The same may be true for proteins near their isoelectric point [35]. Specifically, for SPR biosensors, this occurrence can cause interference, possibly preventing the detection of targets available at low concentrations.

The real-time identification and monitoring of specific analytes circulating in body fluids (such as blood, serum, saliva, urine, or cell lysate) would greatly aid our understanding and advance the development of personalized medicine [36]. Although serum, plasma, or blood are the most appropriate samples for sensing and translation in clinical settings, the optimal goal is to achieve in vitro biosensing running assays using whole blood or other undiluted matrices, with minimal sample preparation [7]. Ideally, the plasmonic sensor should also be validated by performing measurements on unmodified and unspiked samples, comparing results with gold-standard technologies, and, ultimately, be integrated into point-of-care (POC) devices [37].

A serious obstacle, however, is the fouling background associated with proteome present in the blood, which is a very complex matrix with a protein load of 60–80 mg mL^−1^, thereby increasing the possibility of undesired binding to either the sensor surface or the analyte [38]. The unwanted interaction of the analyte with bulk proteins of the biofluids may significantly reduce the effective target concentration due to hindering of its binding site, as demonstrated by the fraction bound to the free prostate-specific antigen (PSA) in serum samples [39], or by the interference in the detection of the chemokine receptor CXCL12 [40], following the binding with glycosaminoglycans found in urine samples from rheumatoid arthritis patients, thus lowering the sensitivity compared to a running buffer solution.

Advantages in antifouling performance can be derived from the reduced number of proteins in different biofluids. For example, the protein content in urine is about three orders of magnitude less than that of blood [41], leading to a less complex matrix with low levels of potentially interfering components in the routine laboratory test. In this direction, the development of analytical platforms for revealing urinary biomarkers provides a feasible tool to overwhelm the intrinsic challenges of traditional blood analysis [42]. In addition, saliva is an attractive biofluid due to the minor complexity of the matrix, which creates less difficulty for non-invasive sampling and sensing, being available quickly, continually produced, and easy to obtain. Unfortunately, not all markers are found in this fluid or correlate to the circulating levels in pathological conditions [43].

High sample-to-sample variability of background response has been reported from different levels of non-specific adsorption observed on the same coating when samples from the blood plasma of different individual donors [44], or serum of infants and adults [45], are analyzed together. Such variability is minimized by using pooled biofluids [46,47]. Furthermore, non-specific adsorption profiles of the SPR response have been reported among type-1 diabetic patients [48]. In this respect, to obtain an accurate compensation for background variations, the adoption of a reference channel [49] that subtracts the signal from non-specifically bound molecules from the total response may help in the initial assessment of the sensor performance, but many real clinical samples behaved differently in the reference channel. The use of a serum sample from a single individual but depleted of the analyte or with the analyte made non-reactive to the SPR sensor has also been proposed. In such a way, the contributions of non-specific fouling and the bulk signal are indistinguishable in both the reference and sensing channels, allowing one to isolate the specific signal [49].

Food sensing is another major application of biosensing, especially for rapid screening of contamination from toxins and pathogens in food samples, since it is necessary to provide real-time results to mitigate foodborne-illness outbreaks. Since contamination of food can occur at any stage of the production, delivery, and consumption chain, food control is paramount to assure high-quality and nutritious food supply while preventing diseases caused by food contamination. The development of new “rapid” detection methods has strongly decreased detection time compared to chemical and microbiological enrichment methods, the most widely used but inadequate [50].

The raw food matrix is a heterogeneous mixture of various components, such as inorganic and bio compounds, which often contain high levels of native microflora. Pathogen and bacterial toxins detection from such a complex sample provide a critical challenge since many matrix components and their intrinsic properties hinder the detection and inhibit enzymatic assays, such as polymerase chain reaction (PCR). Additionally, lipids and various other molecules can interfere with antibody binding, and carbohydrates can interfere with nucleic acid amplification methods [51].

Since most common immunoassays [52] for direct measurement in crude food samples exhibit inadequate sensitivity, cultural enrichment is crucial to give both an opportune cell density to establish the presence of pathogens [53] and adequate cell numbers to have high yield and decent quality in DNA extraction procedures with a sensitivity between 103 and 104 CFU mL^−1^, especially in PCR approaches [54]. When using an SPR sensor [55], the low pH of apple juice interfered with an antibody-based sandwich assay, performed for detecting four species of bacteria pathogens (*Escherichia coli*, *E. coli* O157:H7; *Salmonella typhimurium, S. typhimurium*; *Listeria monocytogenes*, *L. monocytogenes;* and *Campylobacter jejuni*, *C. jejuni)* separately and in combination in apple juice, after changing the pH to 7.4 to rectify that interference. The interference with fat and protein components of broiler meat significantly hindered the SPR immunoassay detection of foodborne pathogens, including C. jejuni, also by testing the biosensor on contaminated chicken [56].

Furthermore, when working with a filtered solution from cucumber and ground beef, SPR detection [57] of *E. coli* O157:H7 was inhibited, most likely due to the non-specific adsorption of carbohydrates, vitamins, and dietary fibers. *E. coli* O157:H7 was also revealed with less sensitivity in ground beef samples due to the intrusion of large numbers of nonpathogenic *E. coli* cells, which arrested the transport of the bacteria to the sensor surface.

Generally, detection limits in complex media are higher than those in a running buffer where sensitivity arrives at femtomolar levels. A dramatic improvement of the detection threshold for toxin activity is obtained through the immunoprecipitation of toxin to remove interfering compounds from sera, as demonstrated by the immunoprecipitation procedure optimized by Ferracci et al. [58] to gain acceptable sensitivity due to the presence of determinants that result in non-specific hydrolysis of substrates by endogenous proteases. Moreover, “masked” mycotoxins that uncover their harmfulness after conjugation with sugar or organic acid are significant targets in food analysis [59].

**Figure 1:**
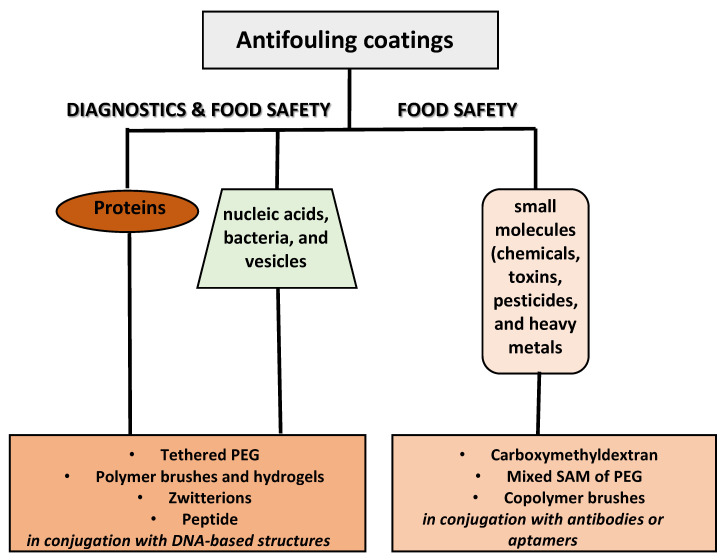
Schematic overview of the various approaches to fabricate antifouling surfaces for diagnostics and food safety.

For all the above considerations, the choice of antifouling coating is crucial for maintaining the analyte’s long-term stability in complex media without altering its quantification. A schematic overview of the various approaches to fabricate antifouling surfaces for detecting different analytes of interest in food and clinical samples is displayed in Figure 1.

## 3. Type of Antifouling Materials

**Figure 2:**
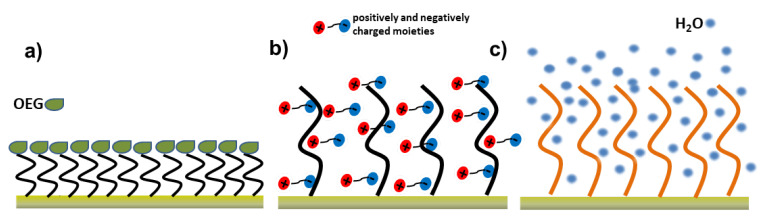
Pictorial illustration of some of the antifouling layers: (**a**) SAM presenting OEG, (**b**) zwitterionic, and (**c**) hydrogel polymers.

A wide range of molecular systems with potential antifouling activity has been identified. According to their chemical composition, they may be classified in alkane thiolate self-assembled monolayers (SAMs) [60], poly (ethylene glycol)/oligo (ethylene glycol) (PEG/OEG)-based materials [61], zwitterionic compounds (with moieties, such as carboxybetaine (CB), sulfobetaine (SB), and phosphorylcholine (PC)) [62], polysaccharides [63], peptides [64], mixed-charge polymers [29], or hydrogels [65] (Figure 2).

The antifouling phenomenon generally involves several cooperative mechanisms such as hydration, steric hindrance, ionic solvation, and charge balance [30,38]. Moreover, physicochemical properties, such as polymer chain flexibility, packing density, and molecular weight, play a critical role in providing the antifouling performance of hydrophilic polymers, which is tightly and primarily correlated with the formation of a hydrate layer that prevents the non-specific adsorption of molecular constituents on the surface. Generally, when neutral coatings are used (e.g., PEG chains), this water layer is tethered through hydrogen bonding [66], while using charged polymers (e.g., zwitterionic), the strong electrostatic component positively affects the thickness of the water layer [67].

### 3.1. Poly (Ethylene Glycol)/Oligo (Ethylene Glycol)-Based Self-Assembled Monolayers

Short-chain alkanethiolated SAMs can self-assemble on the gold surface of SPR sensors to form densely packed and ordered architectures that generate hydration layers. SAMs are typically arranged using alkanethiols with the terminal oligo (ethylene glycol) (OEG) groups, with reactive and functional moieties exposed to the surface. The active functional groups can be brought to the surface before or after the formation of the SAM. The immobilization orientation and efficiency depend on available functional groups and accessible areas of charge localized on the bioreceptor, which are highly variable when considering protein, acid nucleic, lipids, and other components. Since the hydration layers are influenced by the electrostatic interactions established between polymers and those components, the distribution of charge density in SAMs with opposing charges presents better resistance to protein adhesion [4].

Polyethylene glycol (PEG; >10 EG units) and its derivatives such as oligoethylene glycol (OEG; 3−10 EG units) have unquestionably been the most accessible antifouling materials [68]. PEG and OEG layers also enhance the biocompatibility of biosensing by leaving the active functional groups readily accessible for the immobilization of the bioreceptor.

Several mechanisms have been proposed to explain the protein-resistant properties of SAMs with PEG/OEG, owing to the steric hindrance and hydrophilicity generated by the long-chain polymers, with their densely packed structure and stable hydrogen bonds. The widely accepted process is that when unwanted biomolecules from the biofluids approach the PEG/OEG surfaces, there is an entropic loss associated with reduced surface group mobility and flexibility of PEG chains for steric suppression upon fouling, combined with strong hydration of these layers, which contribute to form a physical and energetic barrier that minimize protein adsorption. Typical protein adsorption from single-protein solutions and blood plasma onto SAM surfaces range 100 pg mm^−2^–3000 pg mm^−2^, as measured by SPR [7].

Different surface modification techniques have been used with PEG chains ranging from physical adsorption (coating) to covalent grafting to chemisorption. A random coil conformation without overlapping among chains is obtained when PEG at a low density is covalently grafted or chemisorbed to the surface by its end group. PEG chains cannot maintain the random coil state and enlarge to have an arrangement as the density increases. The chains are wholly extended at a higher density, and the surface is in the “brush regime” [35,69].

The modifications of PEG/OEG chains via changing the length, size, and morphology of the unit, the polymerization density, and the properties of the terminal group have been investigated to improve PEG polymers’ antifouling properties [70,71].

**Figure 3:**
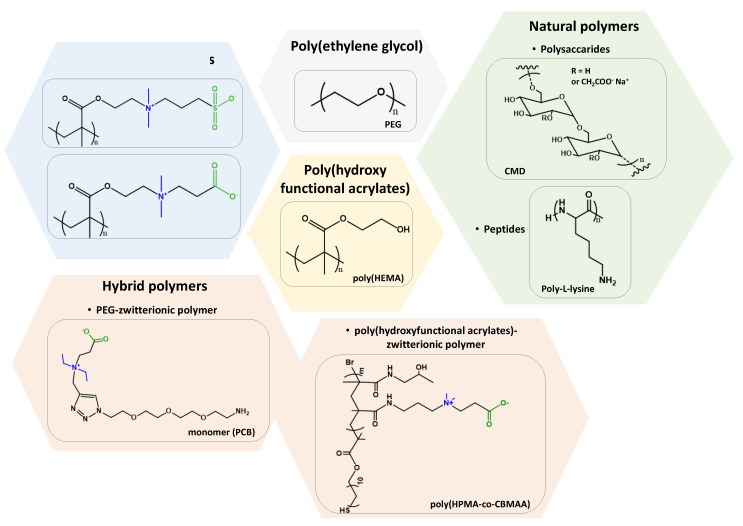
Chemical structure of different layers designed as antifouling coatings.

Despite the potential of PEG compounds as an antifouling agent, the susceptibility to oxidative damages [72] and the requirement of high molecular weight to keep the colloidal stability [14] limit the antifouling capabilities of PEG-based materials in long-term applications. For these reasons, alternate hydrophilic polymers such as zwitterionic compounds, polyamides [73], polydopamines, [74], and naturally occurring polysaccharides have been evaluated for antifouling applications. PEG limitations have led to developing a range of alternative polymeric materials, several of which are shown in Figure 3.

### 3.2. Zwitterionic Surface Layers

Zwitterionic polymers have emerged as promising candidates for advanced antifouling/biocompatible materials because of their high hydration capacity and electroneutrality. They exhibit balanced anionic/cationic groups on their molecular structure, making them highly hydrophilic and antifouling while maintaining overall charge neutrality [75].

A critical factor determining the non-fouling properties of polyzwitterionic materials is controlling both charge distribution uniformity and charge neutrality of two oppositely charged moieties on the surface. Therefore, protein adsorption is largely inhibited when the layer is electrically neutral, as shown by investigating zwitterionic electrolyte layers at different pH values [76]. Such factors can be tuned either by using zwitterionic units [77,78] or, more easily, by mixing positively and negatively charged moieties in mixed-charge self-assembled monolayers (SAMs) [30], polymer coatings [29], or hydrogels [79].

Simple methods for the surface functionalization with zwitterionic compounds via covalent bonds would allow solving the limitations linked to the atom-transfer radical polymerization (ATRP) [80] and surface-initiated photoiniferter-mediated polymerization (PIMP) [81]. Common zwitterionic polymers are based on phosphorylcholine (PC) [82], betaines (CB or SB) [31], and polypeptides/peptoids [83], widely applied to biosensors, thanks to good biocompatibilities and potential antifouling applications. The implementation of 3D-structured zwitterionic CB hydrogels onto the sensor surface has also demonstrated its usefulness for blocking protein fouling or bacterial infections (<5 ng mL^−1^ of foulants in undiluted serum) [84,85].

Densely packed poly (sulfobetaine methacrylate) (polySBMA)-grafted surfaces have also been reported to be utterly resistant to the adsorption of plasma proteins, including human serum albumin, gamma globulin, fibrinogen, and lysozyme, even at low ionic concentrations [86].

Along with poly (SBMA) coatings, carboxybetaine–methacrylate (poly (CBMA)) brushes have been prepared, and both zwitterionic polymer brushes (poly (SBMA) and poly (CBMA)) grafting surfaces reported a reduced adhesion of fibrinogen to a level on par with PEG-based coatings [87]. The high resistance of the plasma protein adsorption from poly (CBMA) and its unique anticoagulant activity make poly (CBMA) attractive because of its unique versatility for immobilizing ligands, such as proteins and antibodies, onto the carboxyl groups.

### 3.3. Natural Compounds and Biomimetic Materials

Naturally occurring biomolecules such as amino acids, peptides, and polysaccharides are also commonly used to develop innovative antifouling materials since they have a protease resistance property, precise control of molecular weight, and broad versatility of the side-chain composition [88]. One theory to explain biofouling prevention through the peptide systems is the structural conformation under physiological conditions [89]. Many examples of antifouling materials based on peptides have been reported in the literature [90,91]. The antifouling performances of polysaccharide-based coatings in both single-protein and complex media are lower than those of PEG or zwitterionic layers, and their use is principally focused on the functionalization of sensing surfaces [92]. Dextran-based hydrogel layers have been employed for decades in typical anchoring procedures to modify SPR sensor surfaces in an extensive range of commercial applications. Lately, a novel strategy implies the exploitation of hyaluronic acid covalently bonded to the sensor chip. The amide and carboxyl groups of the disaccharide form the water layer, providing a unit inert and stable ultralow-fouling surface (3 ng cm^−2^) [63].

Several research groups reported hybrid surface peptide-based modifiers consisting of a poly-l-lysine (PLL) polypeptide backbone partially grafted with different antifouling molecules, like PEG side-chains, through amine residues [93]. PLL is a versatile polymer composed of positively charged lysine amino acid as a repeat unit. Such a polymer exhibits hydrophilicity, excellent biocompatibility, and an acceptable degree of biodegradability [94,95]. The exploration of new architectures or mixed synergic interfaces as antifouling layers is expected to be very important, particularly when considering that these interfaces must perform well diagnostically and be nontoxic in most applications.

Recently, a new functional low-fouling poly-l-lysine (PLL)-based surface layer has been introduced [29,96]. The new PLL-based layer includes a densely immobilized CEEEEE oligopeptide, creating a charge-balanced system preventing the non-specific adsorption of plasma components, and comprises sparsely attached peptide nucleic acid (PNA) probes complementary to the circulating target DNA sequence carrying KRAS mutations from cancer patients.

Poly (hydroxyfunctional acrylates), which include polymers such as poly (2-hydroxyethyl methacrylate) (pHEMA), poly (hydroxypropyl methacrylate) (pHPMA), and poly (*N*-hydroxyethylacrylamide) (pHEAA), are analogues to other PEG alternatives in that they are electrically neutral and hydrophilic. These polymers have a long history as biomaterials. pHEMA hydrogels, for instance, have been commonly used in the area of medical applications, including implants and tissue engineering scaffolds [97].

## 4. Antifouling Strategies for SPR Biosensing in Clinical Diagnostics

At first, the carboxymetildextran (CMD) chemistry [98] was adopted as a hydrophilic and protein-compatible hydrogel to minimize the impact of non-specific adsorption on SPR surfaces and allow increased immobilization as compared to SAM-based coatings. Even if the CMD surface displays positive performance in running a buffer or biofluids diluted more than ten times, it is not enough to get a stable baseline with an undiluted serum or plasma [99].

Other works using a dextran coating showed the influence of pH on the amount of the captured target. The antigen surface charges depend on the isoelectric point, and it varies depending on the pH of the buffer solution. An SPR immunosensor makes use of the antigen as charged species in a mixture including Bovine Serum Albumin (BSA), Prostate-Specific Antigen (PSA), and C-reactive protein (CRP) to reveal PSA [100] in diluted serum samples by tuning the pH of the carrier buffer. Serum samples were first diluted sequentially to 10, 100, 1000, and 10,000 times by adding increasing amounts of PBS buffer at pH 6.5, and they were spiked afterward with PSA solutions at a concentration of 0.01 mg mL^−1^. It has been reported that the quantity of interfering proteins was ~66 times that of the amount of PSA for samples diluted 100 times or more.

Most frequent are SPR sensors that rely on PEG-based SAMs interfaces, as the format design exploits a newly presented multiplex SPR imaging (SPRi) sensor for the parallel detection of three human pancreatic hormones (insulin, glucagon, and somatostatin) [101]. Since the analytes were small peptides, sensitivity was improved through a direct competitive assay by immobilizing different hormones on the corresponding antibody functionalized spots on the surface, modified with a mixed SAM of CH3O-PEG-SH and 16-mercaptohexadecanoic acid, following a mixture of different antibodies. Inhibition of antibody binding due to binding occurring with hormones in the solution leads to a smaller SPR signal with a higher concentration of hormones in solution, and vice versa. Opposite to most reports in the literature [102], the mentioned approach exploits a long-chain polymer as the spacer and a short-chain as the anchor. Although the SPRi sensor displayed high selectivity to the corresponding hormones determined with LODs for insulin (1 nM), glucagon (4 nM), and somatostatin (246 nM) with negligible interference from 1 mg mL^−1^ BSA or lysozyme (LYS), no application to real-life samples is shown.

In contrast, a recent SPR application describes the detection of the main immunogenic peptide of wheat gluten in urine samples from healthy donors and celiac disease patients by exploiting a functionalized PEG/SAM monolayer with an indirect competitive assay [103]. The biosensing strategy benefits from a sound functionalization procedure and a stable biorecognition layer involving the immobilization of the prolamin working group (PWG)-gliadin reference material. The assay exploited two specific monoclonal antibodies to the epitopes of PWG-gliadin peptide that bind to different recognition patterns of the gluten immunogenic peptide (GIP) by enabling sensitive detection from 1.6 to 4.0 ng mL^−1^. The synergic effect of the PEG layer and an extra-blocking step, comprising BSA added at 10 mg mL^−1^ in PBS with Tween for 2 min before each urine analysis, suppresses the interference of the matrix on the assay performance without disturbing the binding of the antibody to the PWG-gliadin layer. The analysis takes less than 15 min without pretreatment, extraction, or dilution, and showed reproducibility, good recovery, and reliable correlation with the standard method for GIP determining in urine occurs.

**Figure 4:**
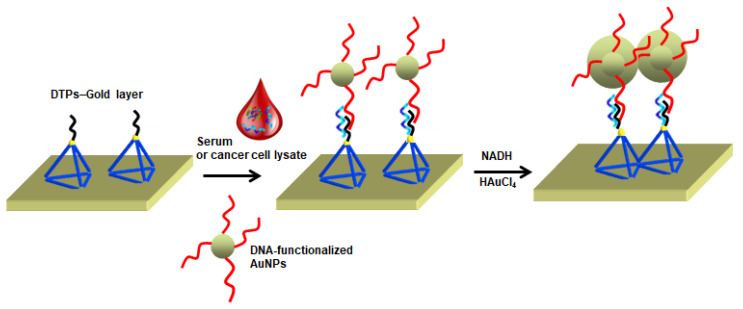
A pictorial illustration of the low-fouling SPR biosensor to detect miRNA in serum or cancer cell lysate based on the DNA tetrahedron probes (DTPs). Adapted with permission from Ref. [104]. Copyright (2018) American Chemical Society.

A very singular approach comprising an antifouling surface, consisting of DNA tetrahedron probes (DTPs) covalently attached to the gold-coated surface of the SPR sensor, enabled sensitive and selective detection of microRNA in undiluted human serum and cell lysate [104]. The excellent antifouling performance even in undiluted serum was accomplished by combining the steric hindrance produced by origami conformation and the strong hydration ability of the tetrahedrons. In this strategy, the SPR signal was enhanced by enlarging DNA functionalized AuNPs (Figure 4). The formation of DTPs-Au coating yielded ultralow adsorption (less than 8.0 ng cm^−2^) using two interfering proteins (myoglobin and HSA) in five complex matrices (100% serum, 100% plasma, 9.85 × 108 red blood cells mL^−1^, 5% whole blood, and cell lysate). The target miR let-7a was detected at femtomolar levels (0.8 fM) and selectively discriminated from a homologous family.

With this perspective, SPR has been used to detect human thrombin with a 3D DNA origami structure modified with an aptamer [105]. The DNA origami allows arranging the thrombin-specific aptamer on the SPR sensor’s surface with nanoscale precision, contributes to favoring the thrombin detection with a broad linear detection range, and highlights the potential of using novel DNA origami-based structures in complex matrices to prevent fouling [106].

Similarly, in the context of programmable DNA structures, a new SPRi biosensor was accomplished for ultrasensitive detection of non-small cell lung cancer (NSCLC)-associated exosomal miRNAs [107]. By synergizing the DNA tetrahedral framework (DTF) as the antifouling layer and Au-on-Ag heterostructure as the amplifier for SPR response, the biosensor works fine in a complex biological fluid by exhibiting excellent sensitivity down to 1.68 fM and a wide dynamic range. Most importantly, the biosensor showed high-throughput capability to detect four exosomal miRNAs in parallel in a single clinical sample.

Contrary to the negatively charged DNA-based antifouling layers mentioned above, a recent non-fouling membrane consisting solely of the positively charged lipid bilayer mimic, 2-dioleoyl-sn-glycero-3-ethylphosphocholine (EPC+), has been investigated [108]. First, the sensor surface was functionalized with a mixed SAM of 3-mercapto-1-propanol (MPO) and protein A. Then, the last layer was used for the successive oriented capture of anti-IgG after having been brought in contact with the non-fouling lipid (EPC+) membrane. Surprisingly, the natural zwitterionic 1-palmitoyl-2-oleoyl-sn-glycero-3-phosphocholine (POPC) membrane presented higher fouling property than the positively charged EPC+ membrane, which was utilized to carry out the detection of immunoglobulin G (IgG) in undiluted mouse serum through an immune-sandwich format via SPR. In addition, the advanced EPC+ interface was successful at reducing non-specific interaction from human blood serum and plasma spiked with cholera toxin (CT), allowing for quantification of targeted antigens (IgG and CT) within these complex matrices.

Several biosensors exploiting peptides’ flexible structures and naturally high biocompatibility have been developed [109]. In one recent study [110], an SPR sensor for detecting a tumor biomarker of platelet-derived growth factor (PDGF-BB) has been performed. To this end, mixed SAMs of the antifouling CPPPP-EKEKEKE peptide and an aptamer covered a home-built gold-coated fiber-optic (FO)-SPR. Aptamer-modified AuNPs and the PDGF-BB peptide produced a layer that was then employed in a sandwich format. In the presence of the PDGF target, the gold film-coated aptamer on the FO-SPR sensor produces a sandwich with free aptamer-modified AuNPs in solution. BSA and LYS proteins were selected as single interfering species to explore the antifouling capability. Analysis of PDGF-BB in 10% human serum showed the method’s high selectivity and sensitivity combined with the low fouling ability of the peptide-based interface and exhibited a linear PDGF detection range from 1 to 1000 pM with a low LOD of 0.35 pM.

**Figure 5:**
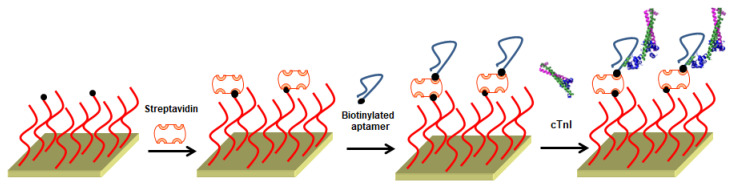
A pictorial illustration of the multistep process used for the cTnI biomarker detection. The peptide-modified gold layer was used for the SPR experiments by first attaching Streptavidin, then immobilizing biotinylated aptamers on the SPR surface. Adapted from Ref. [112].

Out of the outstanding antifouling property of the zwitterionic peptide, a zwitterionic peptide with terminal biotin in its sequence (N’-biotin-EKEKEKE-PPPPC) [111] was used for constructing an innovative aptasensor with high resistance to protein fouling, along with admirable selectivity and specificity towards cardiac troponin I (cTnI) in the presence of complex media [112]. The immobilization of cTnI-specific binding aptamers has been conducted by the streptavidin-biotin system, as shown in Figure 5. The aptasensor displayed a linear dynamic range of 20–600 ng mL^−1^ and an LOD of ~20 ng mL^−1^, making the prognoses for acute myocardial infarction possible.

Few reports evaluate antifouling capability upon functionalizing the polymer brushes, describing their performances in biosensing by using real-world samples. Vaisocherovà et al. [113] reported on the ultra-low fouling coating of antibody functionalized poly (carboxybetaine acrylamide) (pCBAA) and its use for the detection of a protein cancer biomarker (activated leukocyte cell adhesion molecule or ALCAM) in undiluted human blood plasma, demonstrating higher specificity and sensitivity of the pCBAA materials than those of standard OEG-based AT-SAMs [114]. Recently, the same group demonstrated better passivation of the sensing surface based on a zwitterionic pCBAA SPR array that enabled the direct multiplexing of four microRNAs (miR-16, miR-181, miR-34a, and miR-125b) in crude erythrocyte lysates (EL) obtained from human peripheral blood [115]. The pCBAA brush, roughly 13–40 nm in thickness, covalently attaches ~9.8 × 10^12^ biotinylated DNA probes per cm^2^ and showed strong resistance to fouling from EL samples (<2 ng cm^−2^). The detection of microRNA was conducted employing a sandwich-type hybridization in EL with the aid of streptavidin-functionalized AuNPs for enhancing the SPRi signal. The LODs ranging from 0.35 pM to 0.95 pM were established for microRNAs biomarkers spiked in 90% EL. This sensor provides successful PCR-free detection with no need for miRNA extraction or pre-amplification steps. Moreover, the related LODs were improved by diminishing the sensor operating temperature to less than 15 °C to promote the hybridization process, enabling the detection of miRNAs at levels <0.5 pM for diagnosis of myelodysplastic syndrome in clinical EL samples.

A CB-based innovative SPR approach for monitoring the hepatitis B antibodies in clinical saliva takes advantage of angular interrogation of SPR with the enhancement of the optical signal by fluorescence detection (SPFS) [116]. In this context, an antifouling layer based on brushes of poly [(*N*-(2-hydroxypropyl) methacrylamide)-*co*-(carboxybetaine methacrylamide)] (poly [HPMA-*co*-CBMAA]) was synthesized via surface-initiated atom transfer radical polymerization (SIATRP), and then the Hepatitis B antigen was immobilized through EDC/NHS standard coupling method. After saliva exposure, the target antibody is bound, following the addition of a fluorophore-labeled secondary antibody (IgG) allowing for detection via SPFS. This sandwich immunosensor showed good sensitivity and accuracy, distinguishing between positive clinical saliva samples validated with ELISA quantifications in serum samples. Furthermore, the assay presented excellent antifouling properties, by maintaining the features of the interface functionalized with hepatitis B antigen, compared to fouling resistance being considerably impaired for SAM-based architectures previously reported [117].

Lately, the same antifouling polymer brush (poly [HPMA-*co*-CBMAA]) was synthesized on a gold surface via photoinduced single-electron transfer living radical polymerization and then functionalized by including three aptamers for specific direct detection of two particular binding sites of thrombin (exosite I and II) in minimally processed 10% blood. This brush architecture exhibited a limit of detection with HD1 aptamer equal to 0.7 nM, adequate for predicting a thrombotic event and diagnosis of thrombosis [118].

More recently, a triethylene glycol (PEG (3))-pentrimer carboxybetaine (PPCB) coating has been proposed [31] to fabricate a new SPR surface bearing excellent antifouling properties with a thickness of less than 2 nm, towards undiluted and diluted pooled human plasma samples, also demonstrating the applicability in plasma for the SPRI biosensing of human arginase 1, a biomarker over-expressed in plasma of cancer patients.

## 5. Antifouling Strategies for SPR Biosensing in Food Safety

Most antifouling approaches are well studied for complex biological fluids but less explored for food samples containing diverse sets of interfering components, as previously discussed. The detection of food contaminants using SPR-based biosensors is very promising, and in the literature, many reports have been described [119,120]. The different antifouling strategies for applying SPR-based biosensing in food control, discussed in this section, show the urgent need to detect analytes in actual food samples in relevant concentrations that otherwise cannot be achieved without antifouling strategies. Detection sensitivity is typically lower, and functionalization of sensor surface with specific antibodies or aptamers may be needed.

Tran et al. [121] reported for the first time the selection of DNA aptamers against the major peanut allergen protein, Ara h1. The non-specific binding of Ara h1 protein on the biosensor surface was prevented by using a short amine functional alkane chain with six EG repeats anchored onto activated carboxyl groups of 11-mercaptoundecanoic acid SAM. The selected aptamers specifically bind Ara h1 even when other allergen proteins are isolated from peanuts such as Ara h2. The FO-SPR aptasensor tested a diluted candy bar matrix spiked with increasing Ara h1 protein concentrations, where the selected aptamer was first immobilized on a biosensor for isolating the allergen from the food matrix. Next, the FO-SPR sensor was modified with polyclonal secondary antibodies, while AuNPs with protein A were applied to further enhance the signal with an estimated LOD in the buffer to be 75 nM. The decisive advantage of this assay is not requiring any manual handling steps while utilizing universal PEG as building blocks.

To monitor the lysozyme levels, added as an antimicrobial agent during wine-making, Mihai et al. [122] optimized the aptasensor design with SPR detection for the determination of the allergen LYS with high accuracy and sound sensitivity, reaching a detection limit of 0.035 µg mL^−1^ (2.4 nM) in spiked red and white wines. By critically evaluating the sensor’s performance for practical applications, the fouling resistance here was ensured by modifying the gold surface with a thiol-PEG-carboxylic acid layer.

Nielsen et al. [123] utilized a CMD surface for the detection of the mycotoxins deoxynivalenol (DON) and ochratoxin (OTA) at appropriate levels in beer samples using a portable nanostructured SPRi instrument. Since these potentially mutagenic and carcinogenic mycotoxins can be passed from infected barley into malt and ultimately into beer, controlling beer and its ingredients is required to ensure safety to consumers. In a competition immunoassay, these mycotoxins are covalently attached to the 3-dimensional CMD layer on the sensor surface and compete with free mycotoxins in real samples to bind to a specific antibody. In beer samples, the detection of mycotoxins DON and OTA below theoretically safe levels (LOD of 17 ng mL^−1^ for DON and 7 ng mL^−1^ OTA, respectively) was shown to be possible, allowing the application of the assay in-field. The selective capturing of the analyte by the biorecognition element on this sensor surface was followed by the identification of the analyte using an ambient ionization mass spectroscopy approach. This methodology allowed confirmation of the identity of the target analyte (here DON) and the identification of cross-reacting conjugates in beer samples [124].

Using this identical approach, the same group managed the multiplexed detection of the mycotoxins DON, OTA, zearalenone (ZEA), T-2 toxin (T-2), fumonisin B1 (FB1), and aflatoxin B1 (AFB1) in barley samples [125]. While the detection of mycotoxins DON, ZEA, T-2, and FB1 was possible at regulatory limits (LOD of 26 µg kg^−1^ for DON, 6 µg kg^−1^ for ZEA, 0.6 µg kg^−1^ for T-2, 2 µg kg^−1^ for FB1), allowing in-field applications, the sensitivity of OTA and AFB1 detection needs further improvements for screening at regulatory limits (LOD of 3 µg kg^−1^ for OTA and 0.6 µg kg^−1^ for AFB1).

As seen in these studies, matrix-matched calibrations are strongly required, as assays can be influenced by sample matrices, with different effects for diverse analytes. Especially for the barley study, changes in calibration curves in the buffer and the real sample matrix become visible. Even though CMD surfaces are shown to have good antifouling properties, allowing the application of such a surface in food control, the effects of fouling cannot entirely be eliminated.

The dense and branched structure of polymer brushes favors the prevention of protein adsorption while simultaneously allowing a high biorecognition immobilization capacity, making them an ideal candidate for a functional antifouling surface in SPR biosensing for food control. Alles et al. [126] implemented polymer brushes to detect bacteria using an SPR biosensor for the first time. In their study, they compared the antifouling properties of polymer brushes of methoxy- and hydroxyl-terminated oligo ethylene glycol methacrylate (MeOEGMA and HOEGMA) and polymer brushes of 2-hydroxyethyl methacrylate (HEMA) to SAMs of hexa (ethylene glycol) (EG_6_) and di (ethylene glycol) undecanethiol (EG_2_) and carboxy tri (ethylene glycol) undecanethiol (COEG) (EG_2_/COEG). Both SAMs and MeOEGMA and HOEGMA polymer brushes failed to resist the fouling of fresh milk, powdered milk, and powder infant formula (PIF) samples. Only HEMA polymer brushes showed a complete fouling resistance combined with a high biorecognition immobilization capacity. This sensitivity to fouling is two orders of magnitude lower than fouling observed in widely used SAMs. The antifouling effect can be explained by high hydrophilicity, eliminating the hydrophobic effects with lipid components in milk. These results align with a study implementing HEMA brushes to detect molecules in blood plasma [127].

**Figure 6:**
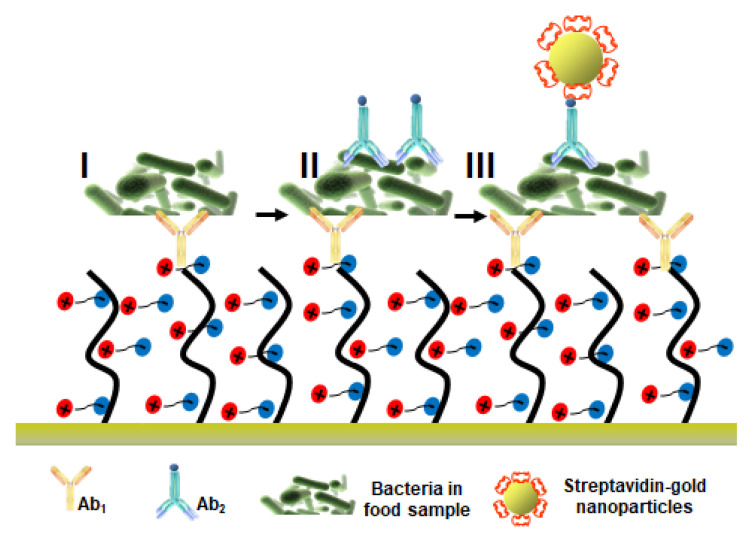
A pictorial illustration of the three-step assay for the detection of bacterial pathogens in food samples. Adapted with permission from Ref. [77]. Copyright (2016) Elsevier.

Homola’s group presents poly (carboxy betaine acrylamide) (pCBAA) brushes as a sensing surface for the multistep detection of bacterial pathogens in crude hamburger and cucumber sample and compares the results to those using a standard low-fouling carboxy-functional mixed oligo (ethylene glycol) alkanethiolate SAMs (AT-SAMs) [77]. Firstly, equal surface functionalization with primary antibodies (Ab_1_) and an equal ability to detect target pathogens in buffer was demonstrated for both surface architectures. Further, it was demonstrated that the pCBAA brushes exhibited a resistance not only toward fouling from food samples, but also to non-specific binding of secondary biotinylated antibodies (Ab_2_) or streptavidin-coated AuNPs (Figure 6).

Fouling levels for pCBAA brushes were up to two orders of magnitude lower when compared to standard AT-SAMs. The sensitivity and selectivity of the pCBAA coated biosensor were extraordinarily high, with detection limits for *E. coli* of 57 CFU mL^−1^ and 17 CFU mL^−1^ for cucumber and hamburger samples, respectively.

These polymer brushes were further improved by the same group when they presented a novel functional coating based on random copolymer brushes combining high antifouling properties of poly [*N*-(2-hydroxypropyl) methacrylamide] (poly (HPMAA)) and high biorecognition immobilization capacities of poly (carboxy betaine methacrylamide) (poly (CMBA)) [128]. The characteristics of these novel brushes were compared to the ones of pCBAA brushes and AT-SAM surface coatings. Firstly, the influence of CBMAA content in poly (CBMAA-ran-HPMAA) copolymer brushes was studied concerning antifouling properties towards different food samples. While all masses of adsorbate remained below 5 ng cm^−2^ (hence classified as ultralow fouling), poly (HPMAA) and poly (CBMAA 7.5 mol %-ran-HPMAA) performed especially well, with fouling falling entirely under the SPR sensitivity. Fouling levels were found to increase considerably after the immobilization of biorecognition elements to the coated surfaces, but the poly (CBMAA-ran-HPMAA) brushes with CBMAA molar contents of 7.5 mol %, and 15 mol % maintained their ultralow-fouling capabilities. When comparing the performance of pCBAA brushes with poly (CBMAA-ran-HPMAA) brushes, it was shown that a poly (CBMAA 15 mol %-ran-HPMAA) brush functionalized with antibodies exhibited better antifouling properties to the extent of two orders of magnitude compared to pCBAA brushes, and even more when comparing to AT-SAM. Very low LODs for various food samples (cucumber, hamburger, lettuce, sprouts) allow applying this polymer brush for food control purposes.

A very different approach for the detection of heavy metals mercury (Hg^2+^), lead (Pb^2+^), and cadmium (Cd^2+^) in contaminated drinking water was presented by Verma and Gupta [129]. Different SPR probes were fabricated with silver (Ag), modified with different coatings (pyrrole, pyrrole/chitosan, pyrrole/ITO, pyrrole/chitosan/ITO), and characterized for their ability to detect Hg^2+^, Pb^2+^, and Cd^2+^ ions. The sensor’s activity relied on the conducting polymer pyrrole/chitosan to bind heavy metal ions over the surface. The pyrrole/chitosan/ITO/Ag-coated fiber optic probe was shown to be sensitive to all of the heavy metal ions and highly sensitive to Cd^2+^. The LOD reported with this sensor was found to be very low (0.129 nM for Cd^2+^, 0.158 nM for Pb^2+^ and 0.293 nM for Hg^2+^). However, the simultaneous detection of heavy metal ions is not possible with this approach.

Table 1 summarizes a range of antifouling polymers applied in SPR biosensors, successfully detecting lower concentrations of different markers in biological and food complex samples because of the minimization of non-specific binding through the design strategies discussed in detail in previous sections.

Table 2 compiles the applications of the best antifouling performances of the different surface coatings described in this review, reported for clinical and food applications, maintaining the ability to analyze biomarkers in plasma or serum and other human fluids (saliva, urine, etc.), as well as food samples (beer, milk, wine, etc.).

## 6. Conclusions

A biosensing device’s functionality depends on the mutual interaction of its fabricated surface with the multi-components of the matrix they are exposed to. This review aims to update an overview of the main representative antifouling strategies and their applications in SPR biosensing, critically discussed together with the great diversity of molecular targets relevant for healthcare and food safety and monitoring (Table 1 and Table 2). We emphasized constructing a low-background and high-sensitivity surface to selectively detect biomolecules of interest in complex samples without interferences from other matrix components.

The significant antifouling capabilities of a range of SAM/PEG or polymer films/zwitterionic brushes have been discussed, as well as the much less investigated human or biological mediums such as cell lysate, saliva, and urine. However, since the content of real-world samples can be relatively complex, no individual antifouling polymer is ideal for all samples. Considering many analytes, assay formats, nanomaterials, and amplification strategies, we are confronted with an almost indefinite number of possible combinations to design antifouling layers for SPR biosensing, with compatible and adjustable characteristics for specific applications. Above all, designing an SPR platform involving antifouling coatings should be properly modulated to enhance its analytical performances while maintaining target activity on the active surface.

In summary, the use of polymer brushes and hydrogels containing hydroxyfunctional acrylates as antifouling polymers has been responsible for the excellent capabilities that the SPR biosensor exhibits in terms of sensitivity, selectivity, stability, and biocompatibility when using real-world matrices involving serum, plasma, saliva, and cell lysate samples. The advance of these biosensors and their exploitation in application fields, such as clinical diagnostics and its translation in personalized medicine, has allowed for reaching the required ultrasensitivity, mainly when DNA nanomaterials have been used jointly as antifouling layers, resulting from a combination of the steric hindrance and strong hydration ability of the DNA-based structures, and labels to enhance the SPR signal. Meanwhile, SPR applications in the food field are substantially improved when antifouling polymers are used in conjugation with antibodies or aptamers. Hence, they can quantify the analyte in a wide variety of matrices and very complex ones, with a sensitivity required by current legislation or below regulatory limits. These applications imply mostly label-free immunosensors, including direct, sandwich, competitive, or indirect format bioassays, enabling the sensitive and selective determinations of the target analytes in a wide variety of food samples through quite simple and straightforward procedures.

## 7. Future Perspectives

It is evident that even if the reported strategies have made significant progress in clinical and food analysis and SPR biosensors have excellent performances for determining relevant analytes in both fields, there is still much to do for their integration in the POC device. Until now, these SPR biosensing methods have only been established but not validated, performing bioassays with a small number of samples often enriched or spiked with the analyte of interest. Additional efforts should address the rational investigation of new antifouling as stimuli-responsive or “smart” polymers [130], with biomolecular switches that reversibly modify their configuration after detecting the specific binding with the target [131], or that experience remarkable physical or chemical changes in response to minor variations in their environment such as temperature, pH, and ionic strength [132].

In the future, efforts should also be addressed to design antifouling coatings for a non-exhaustive series of other candidates in complex fluids like amniotic fluid, breath, feces, aqueous and vitreous humors sweat, and tears. In addition, the single antifouling layer should be helpful for sample matrices with very different physicochemical properties. In this direction, the field of food science remains complex and highly dynamic.

In the meantime, to advance the time- and cost-effectiveness of SPR assays, attempts should be made to accomplish multiplex measurements by performing parallel determination of multiple analytes. Although SPR’s high potential is already recognized, SPR continuous measurements for in vivo assays (or in situ in food control) have not been performed so far.

In addition, efforts should be made to avoid pre-analytical sample processing, signal enhancement, and surface regeneration through multistep procedures. We believe that combining strategies and novel polymer architectures from other biosensors, such as electrochemical and microfluidic sensors or from different fields such as tissue engineering, will pave the way for the next generation of antifouling, stable, and biocompatible materials, and inspire innovative concepts on how to conduct in vivo and in situ measurements of biomarkers.

## Figures and Tables

**Table 1:** Summary of the SPR biosensing approaches aimed at detecting different targets of interest, indicating the antifouling layer used, performances of the SPR sensors in terms of limit of detection (LOD), working range, and matrix type.

Antifouling Layer	Target	Sensing Performance			Ref.
		Operational Range	LOD	Matrix	
carboxymethyldextran (CMD)	DON OTA mycotoxines	60–2000 ng mL^−1^ 10–120 ng mL^−1^	17 ng mL^−1^ 7 ng mL^−1^	beer	[123]
CMD	DON OTA ZEA T-2 FB1 AFB1	26–3200 µg kg^−1^ 13–320 µg kg^−1^ 16–160 µg kg^−1^ 0.6–290 µg kg^−1^ 10–1200 µg kg^−1^ 3–260 µg kg^−1^	26 µg kg^−1^ 3 µg kg^−1^ 6 µg kg^−1^ 0.6 µg kg^−1^ 2 µg kg^−1^ 0.6 µg kg^−1^	barley	[125]
mixed SAM of PEG and 11-mercaptohexadecanoic acid	Peanut allergen Ara h1	0, 211, 423, 634, 846, 1058 nM	75 nM (in buffer)	candy bar	[121]
mixed SAM of PEG and 11-mercaptohexadecanoic acid	LYS	0.05-80 μg mL^−1^	0.035 μg mL^−1^ (2.44 nM)	red and white wines	[122]
mixed SAM of PEG and 16-mercaptohexadecanoic acid	insulin glucagon somatostatin	34–633 ng mL^−1^ 85–1592 ng mL^−1^ 719–4000 ng mL^−1^	1 nM (8 ng/mL) 4 nM (14 ng/mL) 246 nM (403 ng/mL)	pancreatic islet secretome	[101]
ethylene glycol layer and an extra-blocking step comprising BSA	α2-gliadin (33mer) GIP	Using G12 mAB: 3.6 ± 0.2–56.2 ± 13.3 (33mer) 3.4 ± 0.1–35.4 ± 3.0 (GIP) Using A1 mAB: 14.7 ± 3.2–702.3 ± 110 (33mer) 9.1 ± 1.2–172.1 ± 43.3 (GIP)	1.6 ng mL^−1^ (33mer) 1.7 ng mL^−1^ (GIP) 4.7 ng mL^−1^ (33mer) 4.0 ng mL^−1^ (GIP)	100% urine	[103]
DNA tetrahedron probes (DTPs)	miR let-7a	0–2 pM	0.8 fM	100% serum, 100% plasma, 9.85 × 10^8^ red blood cells/mL, 5% whole blood and cell lysate	[104]
DNA tetrahedral framework (DTF)	NSCLC-associated exosomal miRNA-21, miRNA-378, miRNA-200, and miRNA-139	2 fM–20 nM	1.68 fM	10% plasma exosomes in clinical samples	[107]
positively charged lipid bilayer mimic, 2-dioleoyl-sn-glycero-3-ethylphosphocholine (EPC+)	cholera toxin	0, 10, 20, 30 μg mL^−1^	0.05 μg mL^−1^	100% serum or plasma	[108]
mixed SAMs of CPPPP-EKEKEKE peptide	platelet-derived growth factor PDGF-BB	1–1000 pM	0.35 pM	10% human serum	[110]
*N*’-biotin-EKEKEKE-PPPPC	cardiac troponin I (cTnI)	20−600 ng mL^−1^	~20 ng mL^−1^	10% FBS	[112]
poly(HEMA) brushes	Cronobacter	10^8^–10^6^ cells mL^−1^	10^6^ cells mL^−1^	fresh milk powdered milk and PIF samples	[126]
polycarboxybetaine acrylamide (pCBAA)	protein cancer biomarker (ALCAM)	7.8–1000 ng mL^−1^	~10 ng mL^−1^ (unblocked polyCBAA surface) ~100 ng mL^−1^ (blocked COOH/OH OEG)	100% human blood plasma	[113]
pCBAA brushes	miR-16 miR-181 miR-34a miR-125b	0.1–100 pM	0.35 pM (miR-16) 0.39 pM (miR-181) 0.50 pM (miR-34a) 0.95 pM (miR-125b)	crude erythrocyte lysates	[115]
pCBAA brushes	*E. coli* O157:H7 *Salmonella sp*.	1.5 × 10^1^–1.5 × 10^7^ CFU mL^−1^ 2.5 × 10^2^–2.5 × 10^7^ CFU mL^−1^	57 CFU mL^−1^ (*E. coli* in cucumber) 17 CFU mL^−1^ (*E. coli* in hamburger) 7.4 × 10³ CFU mL^−1^ (*Salmonella sp.* in cucumber) 11.7 × 10³ CFU mL^−1^ (*Salmonella sp.* in hamburger)	crude cucumber and hamburger	[77]
copolymer brushes of poly[*N*-(2-hydroxypropyl) methacrylamide] (poly(HPMAA)) and poly(carboxy betaine methacrylamide) (poly(CBMA))	*E. coli* O157:H7	10^4^−10^7^ CFU mL^−1^ (direct *E.coli* detection) 10^2^−10^6^ CFU mL^−1^ (SA-AuNP-enhanced *E. coli* detection)	2.1 × 10^4^ CFU mL^−1^ poly (CBMAA 15 mol %-ran-HPMAA) 81 CFU mL^−1^ poly (CBMAA 15 mol %-ran-HPMAA) in cucumber samples	cucumber, hamburger, sprouts, and lettuce	[128]
poly[HPMA-co-CBMAA]	hepatitis B surface antigen	0.01 and 1 IU·mL^−1^	Not available	10% saliva	[116]
poly[HPMA-co-CBMAA]	thrombin	0–20 nM	0.7 nM (aptamer HD1) 1 nM (aptamer HD22)	10% blood	[118]
triethylene glycol-PEG(3)-pentrimer carboxybetaine (PPCB)	Human Arginase I	0, 12.5, 50 nM	Not available	10% pooled human plasma	[31]
poly-l-lysine (PLL)-mal(26%)-PNA-CEEEEE oligopeptide	KRAS G13D mutated ctDNA	0.5–20 pg μL^−1^	MDC = 0.58 pg μL^−1^ RDL = 1.45 pg μL^−1^	10% plasma from cancer patient	[96]

DON, deoxynivalenol; OTA, ochratoxin; ZEA, zearalenone; T-2, T-2 toxin; FB1, fumonisin B1; AFB1, aflatoxin B1; LYS, Lysozyme; GIP, gluten immunogenic peptides; NSCLC, non-small cell lung cancer; ALCAM, activated leukocyte cell adhesion molecule; FBS, fetal bovine serum; PIF, powder infant formula; MDC, minimum detectable concentration; RDL, reliable detection limit.

**Table 2:** Best antifouling performances of the different surface coatings described in this review, reported for clinical and food applications.

Surface Layer	Clinical Samples							Food Matrices					
	Blood	Serum	Plasma	Urine	Cell Lysate	Saliva	Exosome and Secretome	Beer	Barley	Milk	Wine and Candy Bar	Cucumber and Hamburger	Ref.
CMD								✔ mycotoxins	✔ mycotoxins				[123] [125]
OEG/SAM or PEG- zwitterionic			✔ lung, gastrointestinal and bladder cancers	✔ allergens			✔ hormones				✔ Allergens and additives		[121] [122] [101] [103] [31]
DNA-based structures	✔	✔ breast, lung cancer and hepatocellular carcinoma	✔		✔ breast, lung cancer and hepatocellular carcinoma		✔ colon adenocarcinoma, breast cancer, and lung cancer						[104] [107]
Peptide		✔ sarcomas and glioblastomas, acute myocardial infarction	✔ colorectal cancer										[110] [112] [96]
Zwitterionic brushes	✔		✔ various carcinomas		✔ myelodysplastic syndrome					✔ bacteria		✔ bacteria	[113] [115] [77] [126]
Copolymer brushes	✔ thrombosis					✔ hepatitis B						✔ bacteria	[128] [116] [118]

## Data Availability

No new data were created or analyzed in this study.
